# Multivariate and imaging methods to classify cold tolerance of sugarcane (*Saccharum* spp. hybrids) cultivars and breeding clones

**DOI:** 10.3389/fpls.2026.1882707

**Published:** 2026-08-07

**Authors:** Minori Uchimiya, Collins Kimbeng, Zachary Taylor, Kerstin Höner zu Bentrup, Kaitlyn Taylor

**Affiliations:** 1 United States Department of Agriculture, Agricultural Research Service, Southern Regional Research Center, New Orleans, LA, United States; 2 Sugar Research Station, Louisiana State University Agricultural Center, St Gabriel, LA, United States; 3 Department of Microbiology/Immunology, Tulane University School of Medicine, New Orleans, LA, United States; 4 Oak Ridge Institute for Science and Education Research Program at USDA, Oak Ridge, TN, United States

**Keywords:** abiotic stress resistance, chemometrics, machine learning, precision breeding, sugar crops

## Abstract

Tolerance against winter freeze is the main focus of variety development in Louisiana, which represents the northernmost sugarcane-growing region worldwide. Antifreeze metabolites, xylem structure, and fiber content represent interrelated physicochemical properties contributing to freeze tolerance. This study first classified the cold tolerance of sugarcane cultivars using metabolites in juice as predictor variables. The best-fit model (XGBoost discriminant analysis) estimated the higher cold tolerance of the final on-station clone progeny to the stress tolerance-inducing wild germplasm line. Stalks of the tolerant sugarcane genotype contained higher fiber for mechanical support against cellular injury, compared to susceptible varieties. Fluorescence microscopy visualized phospholipids responsible for maintaining membrane fluidity during frost in lignin surrounding the vascular bundle. Thermal imaging is proposed for real-time monitoring of spatiotemporal temperature changes, as stalk injury is initiated by ice formation at sub-freeze temperatures during winter freeze. As additional datasets for independent prediction become available, developed methods could be used to explore the biomarkers for stress resistance in simpler multivariate discriminant analysis and the distribution of specific biomarkers in cellular components by microscopic imaging, and to trace stalk injury hot spots as a function of time and relationships with resistance markers.

## 1 Introduction

**Figure 1:**
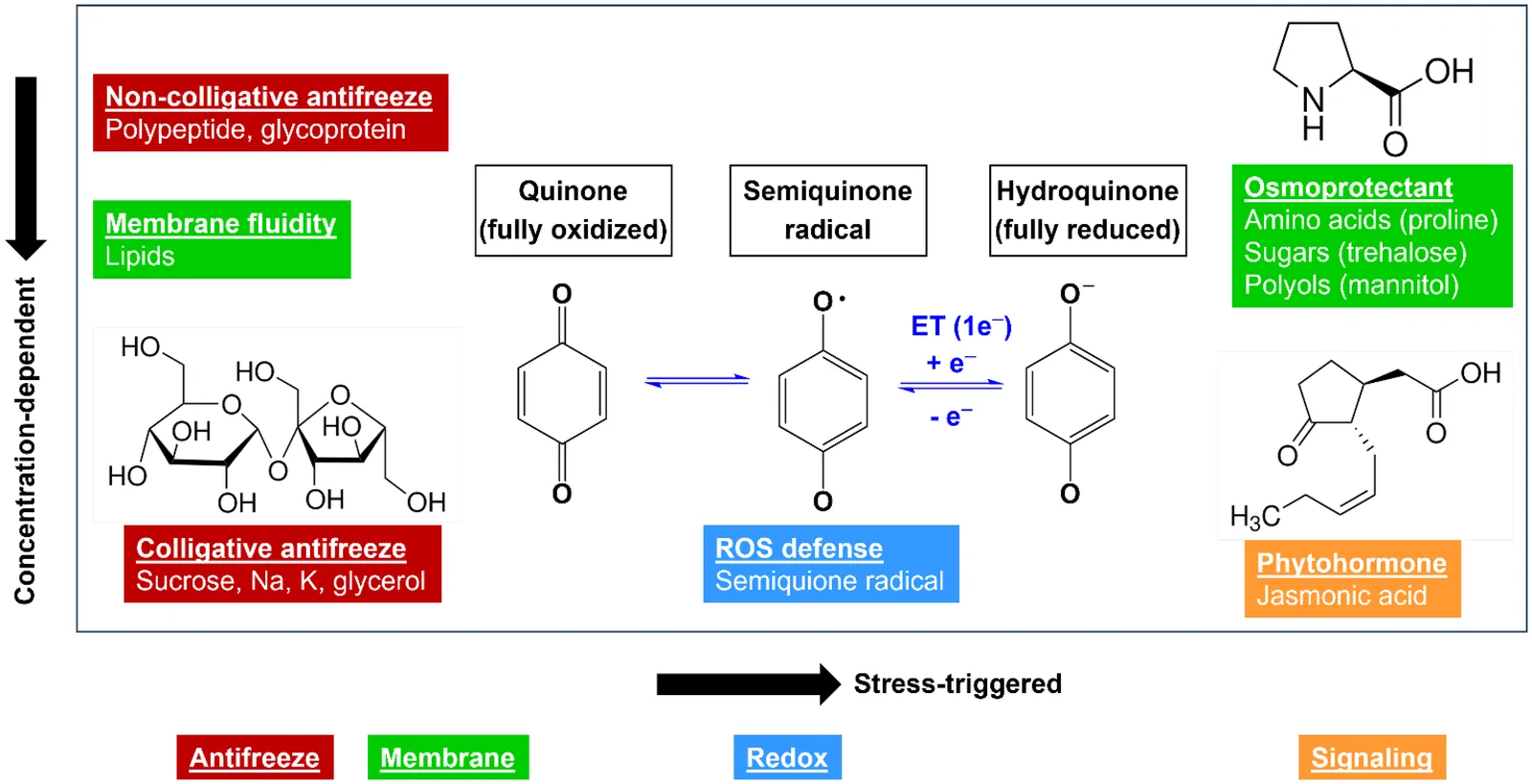
Chemical structures contributing to cold tolerance of sugarcane. Different categories of metabolites are color-coded by the resistance mechanism. Stress-triggered markers (towards right) will increase by frost exposure. Higher concentration dependence leads to lower specificity of the marker inducing stress resistance.

Labor shortage and rising costs are the top challenges the U.S. sugar industry faces today. Cold tolerance is the highest-priority research area for the Louisiana sugar industry, because sugarcane is a tropical crop vulnerable to winter freeze occurring every year especially at the northern border of the state. Released and pre-released sugarcane varieties are intentionally exposed to winter freeze to rate their tolerance by measuring post-freeze changes in juice quality such as theoretical recoverable sugar (Todd et al., 2018). However, no standardized protocol exists to statistically compare the freeze tolerance (rated in arbitrary good, moderate, and poor scales) across sites and years. No genetic or biochemical marker for freeze or other abiotic stress exists for sugarcane in Louisiana. **Figure 1** shows key classes of biochemical players known to induce resistance against water stress such as freeze and drought. Some of those chemical markers could be used to classify abiotic resistance at breeding nurseries and outfield variety test sites through remote sensing (Uchimiya et al., 2025b). Automated and stress marker-guided clone selection methods will dramatically reduce the labor-intensive manual sampling and selection efforts in the current commercial cultivar development program.

The objective of this study was to develop methods to systematically evaluate the stress resistance of sugarcane breeding lines and released cultivars. Partial least squares discriminant analysis (PLS-DA) is widely applied in metabolomics to discriminate classes and to explore predictor variables (metabolites) that are potential biomarkers (Saccenti and Timmerman, 2016). PLS constructs an inverse least squares model using a given number of components to solve a predicted variable from a set of predictor variables. In PLS-DA, predicted/dependent (*y*) variables are “dummies” for the class memberships, while predictor/independent (*x*) variables are the measured metabolite concentrations (Kjeldahl and Bro, 2010). When predictor variables are collinear and contain random noise, variable importance in projection (VIP) scores detect ranges with nonzero regression vector, which is the slope between predicted and predictor variables indicative of proportional and counter-proportional relationships (Uchimiya and Knoll, 2019; Béjar-Grimalt et al., 2025). Machine learning algorithms such as support vector machine and random forest often afford higher accuracy and lower prediction error (without overfitting) than PLS-DA (Béjar-Grimalt et al., 2025) by capturing nonlinear class boundaries without rigid covariance assumption. However, machine learning often requires the iterative optimization of hyperparameters. In addition, the “black box” nature of machine learning limits or hinders model interpretation, compared to VIP score-based interpretation of the PLS-DA model. Only a few studies reported on discriminant analysis (DA) of sugarcane, partly because of its complex genetics and metabolic composition. Sugarcane is highly aneuploid and heterozygous (Meena et al., 2022) and is propagated asexually by clones (Pisaroglo de Carvalho et al., 2014). Sugarcane is a polyploid with varied chromosome numbers and one of the largest genomes among plants (Luo et al., 2023). In addition to its complex genetics, a large biomass of sugarcane leads to competition by height for light, nutrients, and water especially in one- or two-row plots at the early stage of variety selection (Jackson and McRae, 2001; Luo et al., 2023). One available study used VIP scores of PLS-DA to separate the highest and lowest ends of sprouting rates by metabolites in culm of sugarcane (Ferreira et al., 2018). A multivariate analysis on sugarcane reported a separation of leaf metabolites by irrigation for drought-susceptible, but not drought-tolerant varieties (Budzinski et al., 2019). Tyrosine was described as one of the precursors driving the metabolic composition (Ramos et al., 2017). Key metabolites for cold tolerance (**Figure 1**) and underlying mechanisms are described below.

For winter freeze, chemistry of ice formation is central to the tolerance mechanism, as the physical damage of plant tissue is initiated by the ice formation and cellular volume reduction (Storey, 1997). For sugarcane, rupturing injury of stalk by the freeze–thaw cycle initiates dehydration, tissue brittleness, and leakage of unstable juice, causing economic losses in sucrose production (Uchimiya et al., 2025b). Crystallization of ice proceeds through supercooling, nucleation, growth, and recrystallization (Cook and Hartel, 2010). At the recrystallization step, growth of large ice crystals causes dehydration and tissue damage (Worrall et al., 1998). In addition, the freeze–thaw cycle induces xylem hydraulic failure, cavitation, and embolism (Lens et al., 2013; Venturas et al., 2017; Li et al., 2024). A partial cause of embolism is the formation of bubbles from dissolved gas upon freezing. Large bubbles will fill conduits in xylem and cause embolism upon thawing (Nardini et al., 2011). The xylem structure determines hydraulic functions: a wider conduit leads to higher embolism. For example, trees with a wider (>40 μm conduit diameter of xylem) vessel are more susceptible to embolism (Li et al., 2024). Based on microscopy images, the xylem conduit diameter of sugarcane is estimated to range from ≈10 μm in stalks (Uchimiya et al., 2025a) to ≈50 μm in leaves (Rae et al., 2005). Lignin provides a protection against freeze injury by reinforcing xylem conduit walls to resist collapse by internal negative pressure (Venturas et al., 2017). Fiber (cellulose, hemicellulose, pectin, and lignin) contributes to the cold tolerance of tropical crops (Yin et al., 2024) by providing a mechanical barrier against cellular injury from ice nucleation in apoplast (Takahashi et al., 2024). During cold acclimation, cell wall composition and cross-linkage are adjusted to optimize its rigidity (turgor pressure), elasticity (wall stiffness), and porosity (capillary force) (Shikata et al., 2026).

Various metabolites contribute to minimizing the freeze injury described above (**Figure 1**). Lipids are known to maintain the fluidity of membrane (Murata, 1983; Nishida and Murata, 1996), which is the primary site of freeze and dehydration injury (Storey, 1997). Soluble sugars lower the loss of hydraulic conductivity during freeze–thaw cycles by inducing osmotic potential and refilling water into embolism conduits (Li et al., 2024). Malate and glucose are known osmolytes (Andrews et al., 2005). Sugars, proteins, and alkali and alkaline earth metals are known antifreeze agents (Qin et al., 2019). A lower freezing point minimizes the ice formation and cellular volume reduction (Storey, 1997), causing physical injury of the plant from winter freeze. There are two separate categories of antifreeze agents: colligative and non-colligative. Colligative antifreeze, such as sucrose, causes concentration-dependent freezing point depression. Plants need to store a large amount of colligative antifreeze, e.g., as sucrose in sugarcane stalks, because the freeze point constant of water is only 1.86 °C per mole of solute (Duman, 2001). In contrast, non-colligative antifreeze inhibits recrystallization at sub-micromolar concentrations. Protein non-colligatively controls ice nucleation and growth through sorption on ice’s crystal planes. Low concentrations of polypeptide and glycoprotein prevent freeze injury by binding ice crystal facets to prevent crystal growth and recrystallization (Mizuno et al., 1997; Kong et al., 2016).

In a broader context, plants use secondary products (Uchimiya, 2020) as defense phytochemicals or phytohormonal signals against reactive oxygen species (ROS) (Muhlemann et al., 2018) damages caused by water stress including drought (Cia et al., 2012) and high salinity (Wahid and Ghazanfar, 2006). Downregulation of ROS generation was reported for sugar beet taproots under freeze stress (Keller et al., 2021). Because of a shared stress response against ROS, radical quenchers could ultimately be used as a “bulk” biomarker for different types of environmental stress, not limited to the freeze. Organic radicals, especially semiquinones, are a ubiquitous ROS scavenger in nature (Uchimiya, 2020). Semiquinone radicals are stabilized by π–resonance interactions (Uchimiya and Stone, 2009). Spin density is concentrated on the oxygen atoms of hydroxyl substituents, enabling reversible electron transfer (**Figure 1**). As a result, semiquinone radicals can persist under oxic and anoxic environments. When stabilized by complexing metals, half-lives of semiquinones can extend to days. Ubiquitous occurrence of quinones as allelochemicals and other defense phytochemical suggests their involvement in photosynthetic biochemical pathways utilizing the quinone redox cycle.

Our previous study utilized a freeze event in Louisiana to show the correlation between the cold tolerance of commercial cultivars and fluorescent chemical markers (Uchimiya et al., 2025b). Using fluorescence microscopy, Nile red visualized hydrophobic regions in lignin cells around the vascular bundles of sugarcane stalks (Uchimiya et al., 2025a). The vascular bundle is responsible for transporting water and nutrients from leaf to stalk of sugarcane. The xylem structure and its composition influence the freeze susceptibility of sugarcane stalks. However, Nile red is a nonspecific dye for hydrophobic components in the red to yellow emission range. Nile red is used to visualize lipids ranging from polar phospholipids to neutral esterified cholesterol and triglycerides (Diaz et al., 2008). The present study will utilize separate fluorescent dyes each designed to visualize phospholipids, neutral lipids, and lipid droplets to understand which type of hydrophobic component was visualized by Nile red. Once robust multivariate models and more specific microscopy methods are developed, the “net effects” of winter freeze could be evaluated using a thermal camera. The visualization of freeze-induced spatiotemporal temperature changes in sugarcane plants will allow real-time monitoring of stalk injury. Resulting time courses of temperatures and physical injury hot spots could then be used to explore relationships with resistance markers.

## 2 Materials and methods

### 2.1 Field sampling: final on-station nursery clones and commercial cultivars

This experiment is a continuation of our previous report focused on clones at the first-line trial stage (Uchimiya et al., 2025c) of a sugarcane breeding program in Louisiana (Pontif et al., 2023). The first-line stage represents the beginning clonal evaluation stage of 12 yearlong selection activities. The breeding program begins each year by making a few hundred crosses between female and male parents (Pontif et al., 2023). Then, approximately 100,000 seedlings are planted in the field for visual selection, and a few thousand first-ratoon seedling crops are advanced to the first-line trial. At the first-line trial stage, two stalks are planted in a 10-foot, one-row plot for selection by visual scouting and by Brix, and a few hundred plant canes are advanced to the subsequent second-line trial (Pontif et al., 2023). In the second-line trial stage, six clones are planted in a 16-foot, one-row plot for selection by visual inspection and sucrose content in both plant cane and first ratoon. Clones that performed comparable to the commercial checks in the trial are assigned a permanent variety name designation and are planted into three on-station nursery trial locations. The present study focuses on the 31 clones assigned in 2022, along with three commercial checks (L 01-299, L 14-267, and HoCP 14-885) planted into the on-station trial at LSU Sugar Research Station in St. Gabriel, LA (30°16′ N, 90°06′ W). Each clone was planted in a single row (16 feet long) replicated twice. The designated clones and their corresponding parents (30 male and 47 female parents) are listed in **Supplementary Table 1**. Compared to the first-line clones presented previously (Uchimiya et al., 2025c), clones from the final on-station nursery represent a later selection stage and are expected to be more consistent among genotypes. However, neither stage was specifically designed for cold tolerance, although overwintering affects the selection outcomes. The final on-station nursery clones were sampled on 4 December 2024 as the plant cane. Clones were sampled by hand-harvesting 10 stalks for juice extraction and to measure fiber content. The stalks were stripped of leaves and then shredded using a Dedini laboratory disintegrator (Dedini S/A Indústrias de Base). The moisture content (%) of bagasse was measured to estimate fiber content (%) based on stalk weight. One thousand grams of the shredded cane of each clone was weighed and then pressed for 1 1/2 min at 2,500 PSI to extract the juice. The extracted juice was stored at −15°C until analysis (Uchimiya et al., 2023).

A separate set of juice samples was collected for 33 commercial varieties in two replicates on 2 December 2024 at LSU Dean Lee Research Station, Alexandria, Louisiana (31°10′22.7994″ N, 92°24′30.96″ W) as first ratoon. **Supplementary Table 1** provides the reported degree of cold tolerance (−, +, ++, and +++ from susceptible to tolerant), male and female parents, and relevant references for the 33 commercial varieties. This is the second year sampling of the plot, and the fiber content (%) of samples harvested in the previous year (2023) has been presented (Uchimiya et al., 2025c). The fiber content from the 2022–2023 winter freeze trial at a different plot of LSU Dean Lee location (Uchimiya et al., 2025a) was used to validate consistency in cultivar dependence. For each dataset, fiber content was evaluated by Tukey’s pairwise mean comparison (*p* < 0.05) using OriginPro 2025b (OriginLab, Northampton, MA).

### 2.2 Chemical analysis of juice samples

Juice analysis methods had been described in detail (Uchimiya et al., 2023). Briefly, sucrose, glucose, fructose, and *trans*- and *cis*-aconitic acid concentrations were determined using HPLC with refractive index and diode array detectors (Agilent Technologies, Santa Clara, CA). Potassium concentration was measured using a perfection combination potassium electrode (Mettler Toledo, Columbus, OH). Nitrate concentration was measured using an accumet nitrate combination ion selective electrode (Fisher Scientific, Pittsburgh, PA). Electric conductivity (EC), pH, and Brix were measured using dedicated electrodes and a refractometer.

Fluorescence spectra were analyzed by parallel factor (PARAFAC) models (Stedmon and Bro, 2008) with non-negativity constraint using MATLAB version 23.2.0.2459199 (R2023b; Mathworks, Natick, MA) with PLS toolbox version 9.3 (Eigenvector Research, Manson, WA). Consistent with prior reports on Louisiana sugarcane juice (Uchimiya and Spaunhorst, 2020), three fingerprints (98 core consistency) were assigned to tryptophan-like, tyrosine-like, and highly conjugated/aromatic structures. The relative (%) contribution of each fingerprint in a given juice sample was calculated by dividing the intensity of a fingerprint by the total fluorescence intensity of all , and reported as %tryptophan, %tyrosine, and %aromatic.

UV/visible spectra (HP8452A, Hewlett-Packard, Palo Alto, CA) were obtained using a 10-mm path length quartz cell with blank subtraction. Absorbance values at representative wavelengths (228 and 276 nm) were recorded for each sample. In addition, the spectral slope of UV/visible absorbance was calculated following the literature method (Yan et al., 2025). Absorption coefficient [*a*(λ) in 1/m] was calculated as: *a*(λ) = 2.303*(Abs(λ)/*L*), where Abs(λ) is dimensionless absorbance at λ nm and *L* is the cell path length of cuvette. The spectral slope (*S*_λ1-λ2_) was calculated as: *S*_λ1-λ2_ = −((ln *a*(λ_1_)−ln *a*(λ_2_))/(λ_1_−λ_2_)). Two wavelength ranges were selected to calculate slopes: 276–296 nm (S1) and 350–400 nm (S2) based on the literature, and the slope ratio was calculated as S1/S2 (Helms et al., 2008; Yan et al., 2025). UV/visible absorbance decreases exponentially as a function of wavelength, and a decrease in S1/S2 suggests an increase in aromaticity (Helms et al., 2008) of secondary products, e.g., polyphenols in sugarcane juice.

### 2.3 Classification models for cold tolerance of sugarcane

Four different DA algorithms were evaluated using the MATLAB with PLS toolbox: XGBoost gradient boosted tree ensemble for classification (XGBDA), partial least squares (PLSDA), artificial neural network (ANNDA), and ANN deep learning (ANNDLDA). Base algorithms were described in detail previously (Uchimiya et al., 2025b) and are briefly summarized below. Model performance depends on a number of factors including the base algorithm, sample size, and model optimization procedures [variable selection, preprocessing, number of classes, and cross-validation (CV) criteria] (Saccenti and Timmerman, 2016).

PLS (Broadhurst et al., 1997; Hemmateenejad et al., 2002) is a factor-based inverse least squares model, as described in the Introduction (Section 1). A PLSDA model was built using three latent variables with default settings of 0.95 *Q* residuals/Hotelling’s *T*^2^ confidence limits without orthogonalization.

Feedforward ANN with backpropagation neural network (Owens and Filkin, 1989) error correction was employed with two nodes in the first layer and one node in the second layer. Predictor variables were compressed using three principal components, and Mahalanobis distance-corrected scores were used. Default values were used for learning rate (0.125), training cycle iterations (20), maximum training time (20 s), 0.05 terminal root mean square error (RMSE), and 1e−9 RMSE decrease over 100 iterations to end training. ANNDL allows higher numbers (>2) of hidden layers to reduce analysis time for large datasets. An ANNDLDA model was built using the default Scikit-Learn framework, relu activation function, adam solver for weight optimization, 0.0001 L2 penalty, 200 maximum iterations, 12 match size, and one hidden layer.

XGBoost is supervised learning (Chen and Guestrin, 2016) used to select optimal parameter combinations through CV. Variable importance plots were used as a diagnostic tool to evaluate each variable’s importance to the tree ensemble construction as the gain on each node. Compared to decision trees prone to overfitting, the ensemble method of boosting creates more robust models (IBM, 2025). Default XGBDA parameters were used: 0.3 eta learning rate, a maximum depth of six decision trees, 1,000 rounds of tree creation, and 0.5 probability threshold for assigning a sample to a class.

Consistent preprocessing, CV, and class assignment settings were used to compare models. CV employed Venetian blinds with 10 splits and 1 blind thickness. Tolerant (+++) and susceptible (−) classes were used as predicted variables. Autoscale was used to correct for different variable scaling and units by dividing the mean-centered predictor variable by the standard deviation.

The best-fit model was evaluated based on the CV confusion matrix constructed from true positive (TP), true negative (TN), false positive (FP), and false negative (FN) values. For each class (+++ or −), the confusion matrix generates true positive rate (TPR = TP/(TP + FN)) or sensitivity, which is the proportion of correctly identified positive cases; false positive rate (FPR = FP/(FP + TN)) for the proportion of negative cases incorrectly classified as positive; true negative rate (TNR = TN/(TN + FP)) or specificity for proportion of correctly classified negative cases; false negative rate (FNR = FN/(FN + TP)) for the proportion of positive cases incorrectly classified as negative; precision (P = TP/(TP + FP)); F1 score (2*TP/(2*TP + FP + FN) to test model robustness; and number of samples belonging to each class (*N*) (Zhong et al., 2019; Wu et al., 2023). Misclassification error (Err) is the proportion of incorrectly classified samples: Err = 1 − accuracy = (FP + FN)/(TP + TN + FP + FN). A best-fit model reaching TPR, TNR, P, and F1 scores of 1 and FPR, FNR, and Err of 0 was used for all subsequent discriminant analyses.

Once the best-fit algorithm was selected (XGBDA), class prediction probability (Pérez et al., 2009) was calculated for each model by fitting a normal distribution of predicted *y* values. The probability that a sample is class 1 is calculated as: *P*(*y*,1)/(*P*(*y*,1) + *P*(*y*,0), where *P*(*y*,1) is the probability of measuring the given *y* value for a class 1 sample and *P*(*y*,0) is the probability of measuring the *y* value in class 0. If +++, − classes are not adequately separated, the XGBDA model was optimized by iteratively selecting the highest score gain predictor variables.

Three separate models were built to (1) select the best-fit algorithm and test classification using commercial varieties with known cold tolerance (sampled in 2024), (2) independently test the model using the same commercial varieties sampled in a different year (2023), and (3) estimate the tolerance of final on-station clones by CV of a combined dataset for cultivars and clones sampled in 2024. The combined dataset provides a validation of tolerance levels using cultivars, and new assignments for clones with unknown levels of tolerance (Uchimiya et al., 2025c). To account for maturity effects (Uys et al., 2007), only the December sample of each dataset was used to train and test models.

First, a CV model was built on 33 commercial varieties (parents in **Supplementary Table 1** with known levels of cold tolerance) sampled in replicate plots at LSU Dean Lee in 2024. Tolerant (+++, *n* = 10) and susceptible (−, *n* = 18) classes were used as predicted variables. A total of 28 predictor variables included organic (fructose, sucrose, glucose, and total sugar in g/L and *trans*- and *cis*-aconitic acid in mM) and inorganic [K, Na, Ca, and nitrate in ppm, K/Na (M), K/Na (ppm), and Na/K (M)] solutes in juice, fluorophores (tyrosine, tryptophan, aromatic, %tyrosine, %tryptophan, %aromatic, and total fluorescence), UV/visible parameters (228 and 276 nm absorbance, S1, S2, and S1/S2), EC, pH, and Brix.

Next, independent prediction of cold tolerance used the previous year’s (2023) samples as the test set on the model built using 2024 samples. Samples were collected from the same plot in early December of each year, but was plant cane in 2023 and first ratoon in 2024. Because not all parameters in 2024 samples were measured in 2023 samples, the number of variables for this model was reduced to 18: tyrosine, nitrate, %aromatic, K, fructose, pH, %tyrosine, glucose, tryptophan, %tryptophan, total fluorescence, 276 nm, EC, aromatic, *trans*-aconitic acid, total sugar, sucrose, and Brix. Tolerant (*n* = 5) and susceptible (*n* = 9) class members were reduced by half in the combined dataset, because replicate samples were available in 2024 but not in 2023.

Finally, a third model was built on a combined dataset from 2024 sampling: commercial varieties with known tolerance and final on-station clones with unknown tolerance (**Supplementary Table 1**). The cold tolerance of clones was estimated by CV for tolerant and susceptible groups.

### 2.4 Fluorescence microscopy

Detailed fluorescence microscopy procedures including sample preparation, image collection, and analysis were described (Uchimiya et al., 2025a). Briefly, stalk samples were frozen in optimal cutting temperature (O.C.T.) embedding media (Scigen Tissue-Plus, Thermo Fisher Scientific, Waltham, MA) at −80°C and cryosectioned (60 μm thick; CM1860; Leica Biosystems, Deer Park, IL). The O.C.T. medium was allowed to thaw on the slide (Superfrost Plus Microscope Slides; Fisher) prior to staining. The following histochemical staining reagents were evaluated: Evans blue (50% in ethanol), fluorescence brightener 28 (F28, 1 g/L in DDW), LipidGreen2 (LG2, 2.6 μM in DMSO) (Chun et al., 2013), 4-Di-16-ASP (4-(4-(Dihexadecylamino)styryl)-N-methylpyridinium iodide) (DiA, 1 g/L in DMSO), and BioTracker NIM-7 lipid and lysosome live cell dye (NIM7 in DMSO). F28 is a nonspecific stain for cellulose and other cell wall components. Evans blue is often used as a counterstain to diminish blue autofluorescence. Evans blue and F28 were mixed at a 1:1 volume ratio. All other stains were used alone (without mixing with other dyes) and without counter-staining. The cryosections were stained for 5–20 min by fully covering the sample with the stock solution of staining reagent. Stained sections were embedded in VECTASHIELD antifade mounting medium (Vector Laboratories, Newark, CA) before placing a cover glass (0.13–0.16 mm, #1 thickness). The slide was allowed to cure, sealed with clear nail varnish, and stored at −80°C.

Slides were imaged using an inverted widefield microscope (Nikon Eclipse Ti2; Nikon Instruments, Melville, NY). Four filters were used in blue (DAPI), green (GFP), and red (Cy5 and Texas red) emission wavelength ranges. Those filters had the following wavelengths [center wavelength/bandwidth (range) for Ex and Em] and dichroic mirrors (DMs) in nm: 340–380 Ex/435–485 Em, 415 nm DM (DAPI), 460–500 Ex/510 Em, 505 nm DM (GFP), 540–580 Ex/600–660 Em, 600 nm DM (Texas red), and 590–650 Ex/663–738 Em, 655 nm DM (Cy5). Imaging software (NIS-Elements AR, Nikon) was used to acquire, process, and visualize images. The look up table (LUT) function was used to adjust the contrast and brightness of the captured image. Once the image of each filter was optimized using LUT, a composite image was formed to overlay all four filters in an image, except for LG2 images presented as the overlay of DAPI and GFP (but not red filters) to enhance green. Post-processing of images with an on-board deconvolution algorithm was performed to deblur and resolve image details. Red, green, and blue (RGB) values were measured using the color picker and edit color functions of Microsoft Paint. A parameter indicative of red-to-green shift was used for quantitative comparison of different dyes: (G-R)/G (Caraballo et al., 2021).

### 2.5 Thermal imaging

Thermal images were collected using a FLIR E6 handheld camera and processed with the FLIR tools software (Teledyne FLIR, Wilsonville, OR). The camera has a focal plane array uncooled microbolometer for detection at 7.5–13 μm, 150 × 120 pixels infrared resolution, <0.06 °C thermal sensitivity, 45° × 34°field of view, and 0.5 m minimum focus distance. Reflected apparent temperature was measured using crumbled aluminum foil (Costa et al., 2013). Representative images were obtained for frozen and thawed (20 min at room temperature) hand-cut stalks of HoCP 09–804 and L 12–201 varieties at 0.95 emissivity. Stalks were artificially frozen (−15°C), and an image of frozen stalk was obtained on white paper placed on a laboratory bench. Stalks were allowed to thaw at room temperature, and images were collected after 20 min.

## 3 Results and discussion

### 3.1 Discriminant analysis for cold tolerance

**Figure 2:**
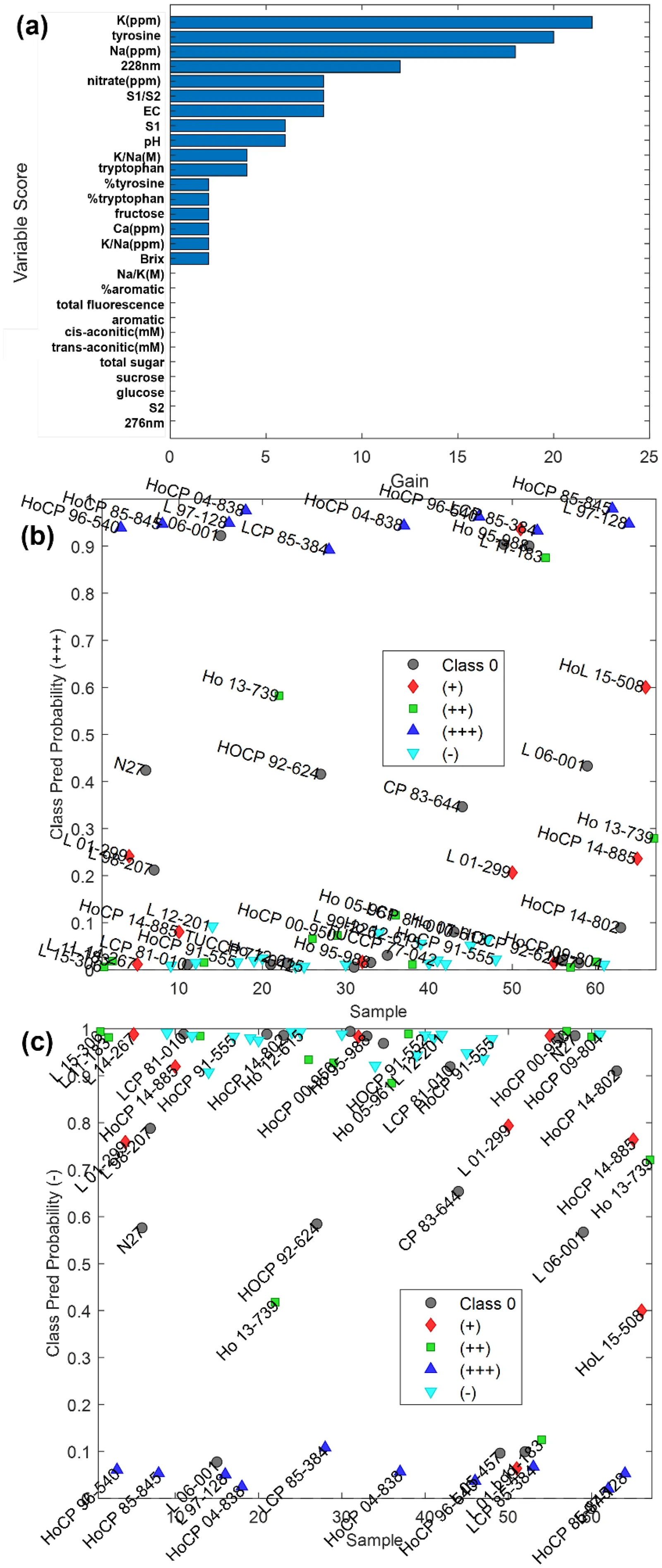
Variable scores **(a)** and tolerant [**(b)**, *n* = 10 including duplicate samples] and susceptible [**(c)**, *n* = 18] class prediction probabilities for XGBDA cross-validation of released cultivars sampled in 2024 at LSU Dean Lee location. A confusion matrix is provided in **Table 1**.

**Table 1** compares the CV confusion matrix for four different algorithms. **Table 1** indicates the robustness of the XGBDA model, consistently for tolerant and susceptible classes. Compared to all other algorithms considered, XGBDA afforded higher TPR, TNR, P, and F1 values and lower FPR, FNR, and Err values. **Figure 2** presents variable scores (a) and tolerant (b) and susceptible (c) class prediction probabilities by CV of cultivars sampled at LSU Dean Lee location in 2024 using the best-fit model (XGBDA, confusion matrix shown in **Table 1**). In **Figure 2a**, variables with the highest gains are both inorganic (potassium, sodium, and nitrate concentrations in juice and electric conductivity) and organic (UV absorbance at 228 nm, S1/S2 attributable to the aromaticity of secondary products, and tyrosine-like aromatic fluorophore) parameters. For “black box” machine learning algorithms including XGBoost (Hu et al., 2023), higher variable scores in **Figure 2a** cannot be directly related to the biochemical origin of tolerance, as described in the Introduction (Section 1). The XGBDA model is able to classify replicates of tolerant standard (HoCP 04-838, blue triangles in **Figure 2b**) in the tolerant class and susceptible standards in the susceptible class (cyan nabla in **Figure 2c**). In **Figures 2b, c**, gray circles (Class 0) are cultivars of unknown cold tolerance. Class prediction probability (*y*-axis) provides an estimate for the tolerance of the genotypes that have not been evaluated for cold tolerance.

**Table 1:** Cross-validation confusion matrix-based comparison of four different discriminant analysis algorithms.

Model	Class	TPR	FPR	TNR	FNR	*N*	Err	*P*	F1
XGBDA	**+++**	0.70	0.11	0.89	0.30	10	0.18	0.78	0.74
	**−**	0.89	0.30	0.70	0.11	18	0.18	0.84	0.86
PLSDA	**+++**	0.40	0.22	0.78	0.60	10	0.36	0.50	0.44
	**−**	0.78	0.60	0.40	0.22	18	0.36	0.70	0.74
ANNDA	**+++**	0.40	0.44	0.56	0.60	10	0.50	0.33	0.36
	**−**	0.56	0.60	0.40	0.44	18	0.50	0.63	0.59
ANNDLDA	**+++**	0.30	0.33	0.67	0.70	10	0.46	0.33	0.32
	**−**	0.67	0.70	0.30	0.33	18	0.46	0.63	0.65

XGBDA was determined to be the best-fit model based on higher TPR, TNR, P, and F1 (reaching 1) and lower FPR, FNR, and Err (reaching 0) for both tolerant and susceptible classes for 33 cultivars sampled in 2024 (**Supplementary Table 1**).

**Figure 3:**
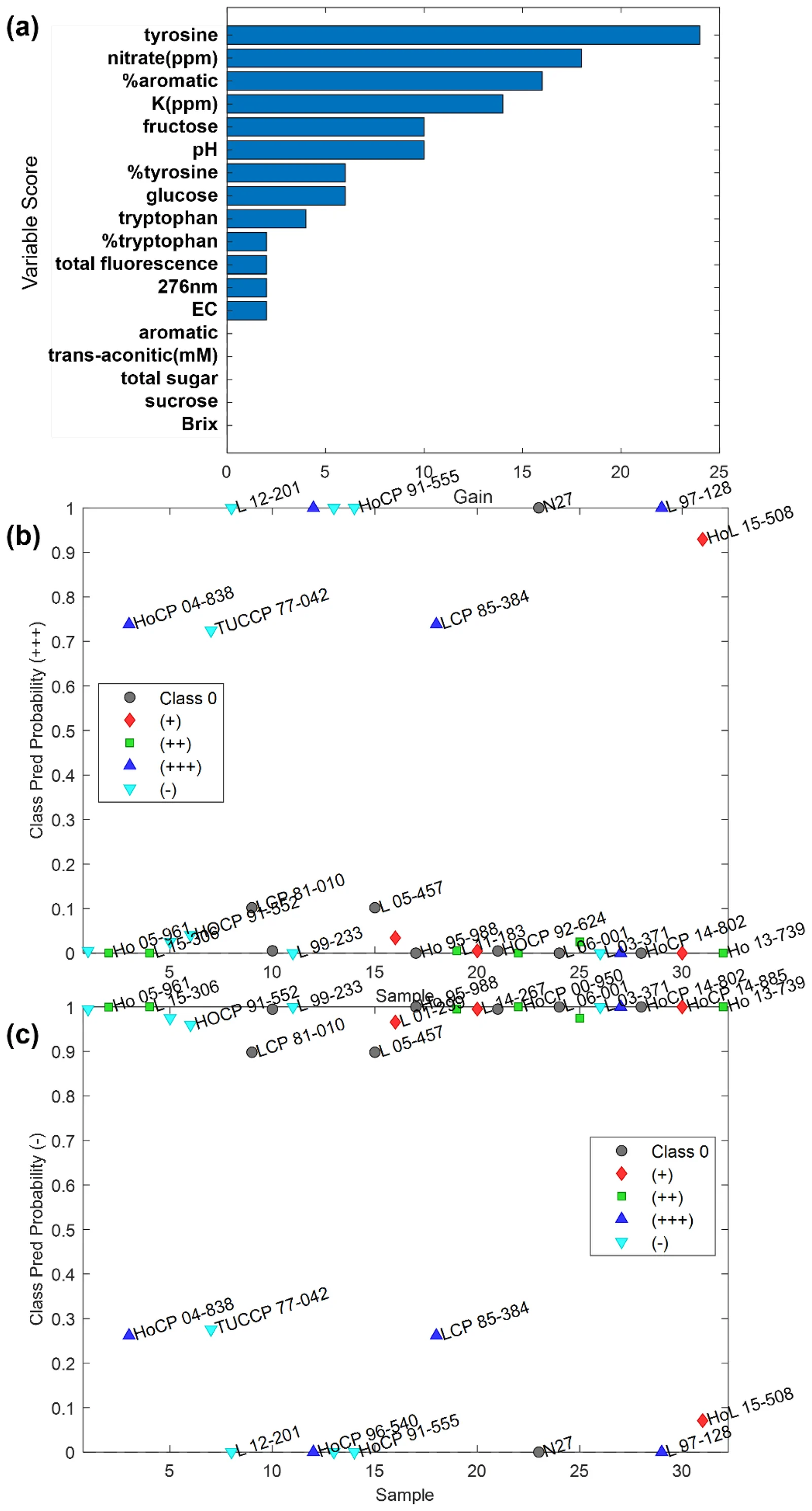
Variable score gains **(a)** and +++ (*n* = 5), − (*n* = 9) prediction probabilities **(b, c)** for XGBDA trained on 33 commercial varieties harvested in 2024 and independently tested on the same sugarcane plants harvested in 2023 at LSU Dean Lee location. Only the top two variables (tyrosine and nitrate) were used for cross-validation and prediction. A confusion matrix is given in **Table 2**.

**Figure 3** shows an independent prediction of tolerant (+++, *n* = 5) and susceptible (−, *n* = 9) genotypes using test sets sampled in a different year (2023). Many of the published classification analyses only presented scores and loadings, omitting model validation and independent prediction (Kjeldahl and Bro, 2010). CV (**Figure 2**) uses a subset of calibration data to test the XGBDA model. **Figure 3** is used to independently test the trained model using a new dataset. Model performance was optimized when the top 2 predictor variables (tyrosine and nitrate in **Figure 3a**) were selected from 18, as shown in **Table 2**. **Figures 3b, c** show prediction probabilities using only the top two predictor variables: tyrosine and nitrate. Comparison of **Table 1** (for XGBDA model) and **Table 2** (using only nitrate and tyrosine as predictor variables) indicates the lower classification performance of independent prediction (**Table 2**) than CV (**Table 1**). The observation is reflected as misclassification of susceptible variety as tolerant (e.g., L 91–555 in **Figure 3b**), and lower-class prediction probability of susceptible variety TUCCP 77–042 in **Figure 3c**. Independent prediction on new datasets generates larger errors than CV on the subset of data used to train the model (Uchimiya and Knoll, 2018). Our previous report provided a detailed discussion on model generalizability and transferability problems and mitigation techniques (Uchimiya et al., 2025b). Data imbalance (*n* = 5 for +++, *n* = 9 for −) causes misclassification of minority class samples (+++ in **Figure 3**) in the majority class (−) (Gupta et al., 2026). When four misclassified samples in the − class (**Table 2**) were removed and classes are balanced (*n* = 5 for both classes), the model performance improved, reaching F1 ≈ 0.9 for both classes in **Table 2**. Misclassification originates from the small sample size, data imbalance, and subjective (visual scouting) method used to rate the cold tolerance of sugarcane (**Supplementary Table 1**). The misclassified tolerant cultivar HoCP 85–845 was also an outlier among tolerant genotypes in previously reported principal component analysis (Uchimiya et al., 2025c). HoCP 85–845 is used as a tolerant check for Mexican rice borer, whereas HoCP 04–838 is used as a susceptible check (Wilson et al., 2015). HoCP 04-838, HoCP 85-845, and L 01–299 are all resistant to sugarcane borer (Salgado et al., 2021). The high fiber content of HoCP 85–845 putatively contributed to pest resistance (White et al., 2008).

**Table 2:** Confusion matrix for independent prediction in **Figure 3**.

Predictor	Class	TPR	FPR	TNR	FNR	*N*	Err	*P*	F1	Misclassified
Top 2^a^	**+++**	0.80	0.00	1.00	0.20	5	0.10	1.00	0.89	HoCP85-845
	−	1.00	0.20	0.80	0.00	5	0.10	0.83	0.91	
Top 2^a^	**+++**	0.80	0.44	0.56	0.20	5	0.36	0.50	0.62	HoCP85-845
	−	0.56	0.20	0.80	0.44	9	0.36	0.83	0.67	HoCP91-555, L12-201, TUCCP77-042, Ho07-613
All 18^b^	**+++**	0.80	0.67	0.33	0.20	5	0.50	0.40	0.53	
	−	0.33	0.20	0.80	0.67	9	0.50	0.75	0.46	

Prediction error decreased by variable selection (from all 18 to top 2) but is higher than cross-validation error (XGBDA in **Table 1**). Prediction performance of the model is further improved when misclassified samples in the susceptible group were removed to balance data (*n* = 5 in both classes).

^a^ Nitrate (ppm) and tyrosine. ^b^**Figure 3a**.

**Figure 4:**
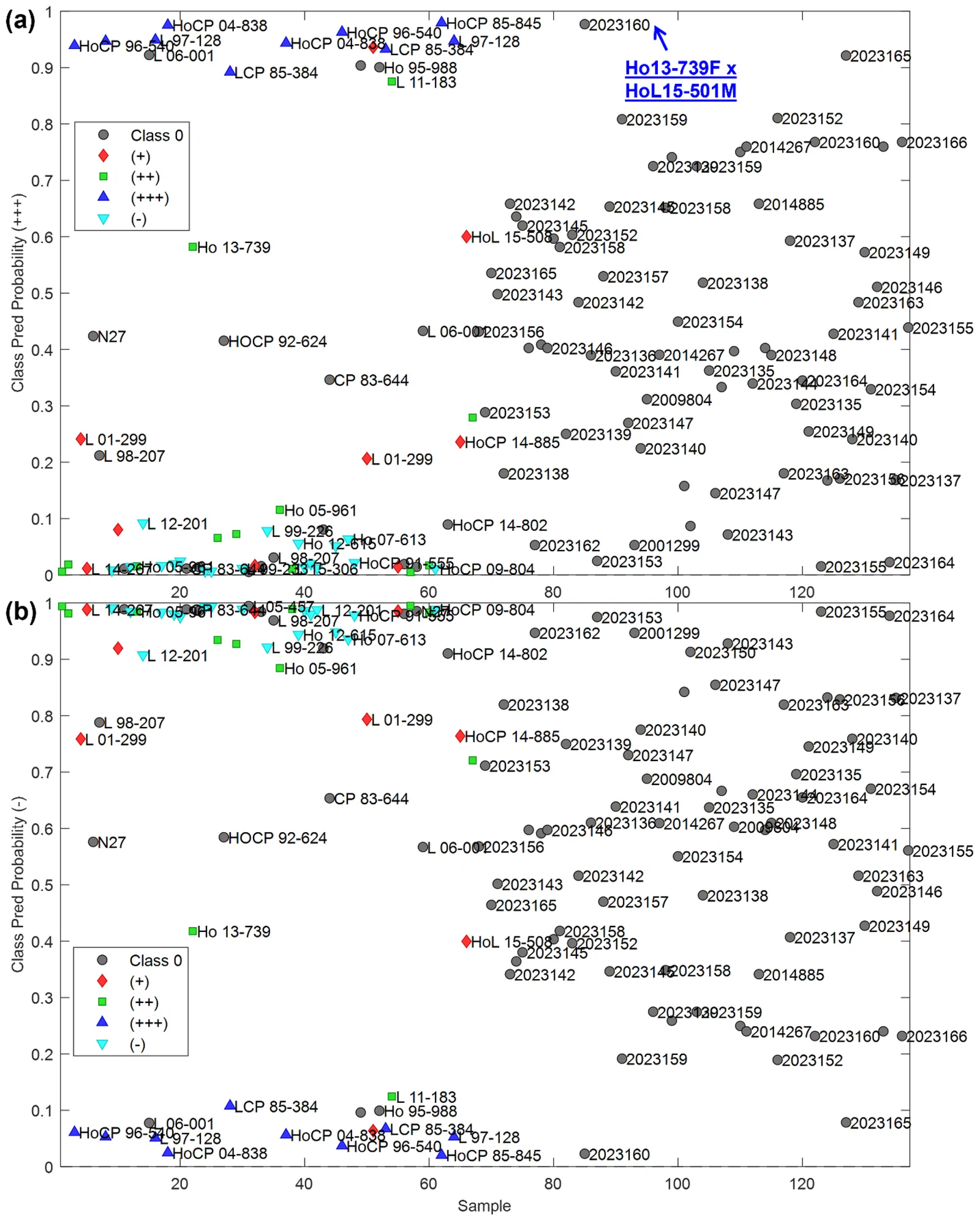
XGBDA for 2024 cultivars in Dean Lee combined with 2024 final on-station clones in St. Gabriel. Tolerant **(a)** and susceptible **(b)** classes were cross-validated by cultivars of known tolerance levels (colored symbols; variable score gains in **Figure 2a**) and applied to clones (gray circles with number-only labels) for estimating the tolerance levels.

**Figure 4** shows the XGBDA-based estimate of cold tolerance for final on-station clones by CV. A confusion table for **Figure 4** is provided in **Table 1** for XGBDA. The model was first cross-validated using tolerant (+++, blue triangles in **Figure 4a**) and susceptible (−, cyan nabla in **Figure 4b**) cultivars to confirm classification robustness. All gray circles (number-labeled without letters) on the right-hand side (*x*-axis above ≈60) of **Figures 4a, b** are clones. Cold tolerance of clones was estimated by CV for tolerant (**Figure 4a**) and susceptible (**Figure 4b**) classes. In **Figure 4a**, the clone predicted to be most tolerant (L 23–160 labeled “2023160” highlighted with blue arrow) has Ho 13–739 as female parent (++). Ho 13–739 has HoCP 04–838 as male parent and the experimental borer-resistant line Ho 06–9610 as female parent. Both of these genotypes arose from crossing with wild germplasm specifically to bring in biotic and abiotic stress tolerance. The male parent of L 23–160 is HoL15-508, which is a cross between CP 83–644 and L 01-283.

**Figure 5:**
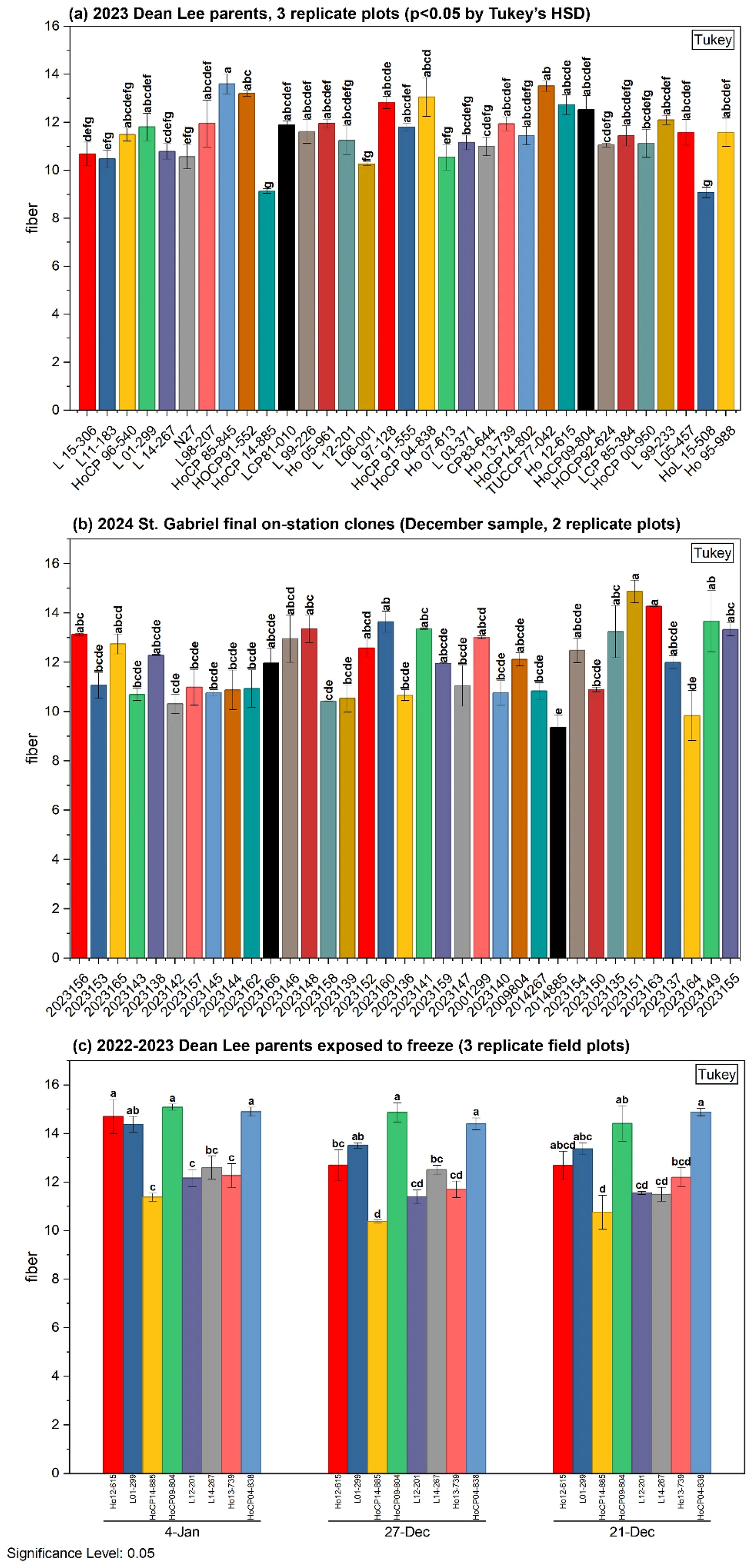
Fiber contents (%) in commercial varieties **(a, c)** and clones **(b)**. Different letters indicate significant difference by Tukey’s HSD comparison (*p* < 0.05) for each panel **(a, b)** and sampling day **(c)**.

Our previous study showed a correlation between fiber content and cold tolerance in commercial varieties (Uchimiya et al., 2025a). Tolerant (HoCP 04-838) and susceptible (L 12-201) varieties were among those with the highest and lowest fiber, respectively (Uchimiya et al., 2025a). High fiber content has also been attributed to the pest resistance of the HoCP 85–845 variety (White et al., 2008). Fluorescence microscopy visualized lipid-like markers in lignin cells around the vascular bundles of the tolerant HoCP 04-838 (Uchimiya et al., 2025a). **Figure 5** examines the relationships between fiber content and tolerance. Section 3.2 will explore the association with lipid markers in fiber (lignin) of sugarcane stalks. **Figure 5a** shows fiber contents (%) of commercial varieties used as the test set in **Figure 4**. Different letters above each bar indicate significant difference (*p* < 0.05 by Tukey) in each panel of **Figures 5a, b** and for each sampling day (21 December 2022, 27 December 2022, and 4 January 2023 from right to left column groups) in **Figure 5c**. In **Figure 5a**, tolerant varieties HoCP 85–845 and HoCP 04–838 were among those with the highest fiber content. HoCP 14–885 and HoL 15–508 had the lowest fiber content, and those cultivars were rated to have lower cold tolerance (+ in **Supplementary Table 1**). Those cultivar trends in fiber content are maintained in samples collected in a different year from a different plot, regardless of sampling days in **Figure 5c**: the highest fiber content for HoCP 04-838 (+++ tolerance) and the lowest for HoCP 14-885 (+). However, susceptible cultivar Ho 12-615 (−) also had high fiber content in both **Figures 5a, c**. In summary, the cold-tolerant standard (HoCP 04-838) had the highest fiber content among the 33 commercial varieties tested in **Figure 5a**. The L 23–160 clone estimated to be cold tolerant (2023160 in **Figure 4a**) has a high fiber content in **Figure 5b**. In contrast, the clone 2023164 estimated to be susceptible in **Figure 4b** had the lowest fiber content in **Figure 5b**. The 2023164 clone is a cross between LCP81–010 female and Ho11–9406 male parents. Based on the available information about those genotypes, both were experimental clones, and LCP81–10 had a high cane yield but low sucrose.

### 3.2 Visualization of lipids in stalk fiber by fluorescence microscopy

**Figure 6:**
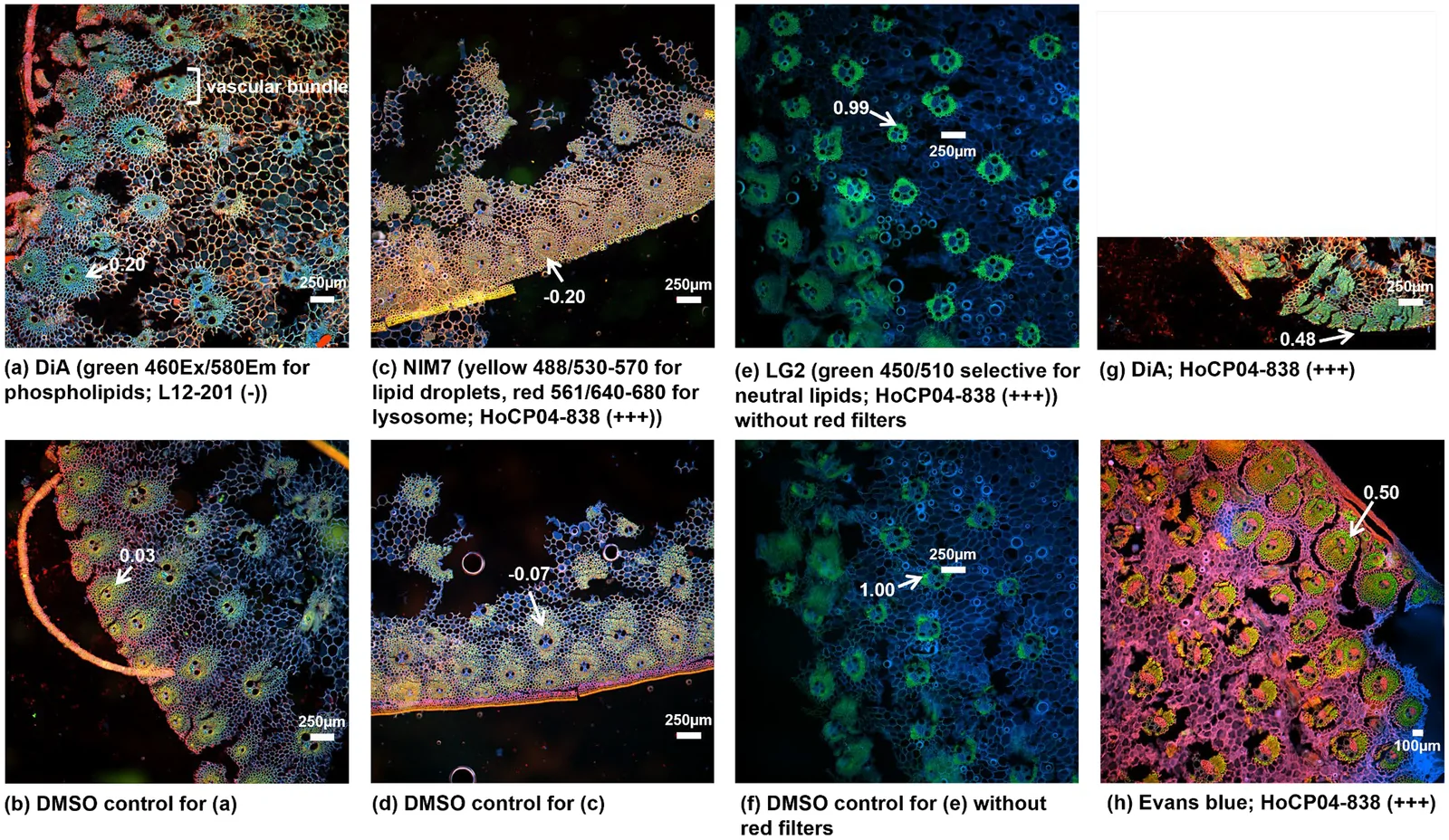
Comparison of fluorescent dyes specific to phospholipids **(a)** lipid droplets **(c)**, and neutral lipids **(e)**. Corresponding controls without dyes are shown underneath **(b, d, f)**. Evans blue is shown as an example of nonspecific dye **(h)**. Tolerant variety (HoCP 04-838) was imaged in **(c–h)**. For DiA **(a, b)**, susceptible variety (L 12-201) is also shown to supplement the fragmented HoCP 04–838 sample **(g)**. All images are presented as deconvolved composite for higher red (Cy5), blue (DAPI), green (GFP), and lower red (Tx red) emission filters, except for LG2 **(e, f)** without red filters to enhance green. Arrowed values in each image provide quantitative comparison of green shifts calculated as: ((G − R)/G).

**Figure 6** shows vascular bundles in sugarcane stalks imaged using dyes specific to phospholipids (DiA in a), lipid droplets (NIM7 in c), and neutral lipids (lipid green in e). For each dye, background (samples in DMSO solvent without dyes) autofluorescence is shown underneath. In (h), Evans blue is shown as an example nonspecific dye staining the entire cellular components. Tolerant variety (HoCP 04-838) is used in **Figure 6** except in panels a and b, where L 12–201 is presented because HoCP 04–838 fragmented (g) during slide preparation. Each image is presented as a deconvolved composite for Cy5, DAPI, GFP, and Tx red filters, except for LG2 (e, f) using only DAPI and GFP to enhance green. The arrowed point in each image shows the green-shift value ((G-R)/G) for stained vascular bundles. **Figure 6a** shows blue/green fluorescence emission (0.20 green shift) of the vascular bundle in the susceptible variety, L 12-201. Compared to yellow fluorescence in DMSO background (0.03 in **Figure 6b**), DiA caused a green shift in **Figure 6a**. Enhanced green by DiA staining is attributable to phospholipids in the vascular bundle. The vascular bundle of the tolerant variety (HoCP 04–838 in **Figure 6g** showing a portion of fragmented sample) is further green-shifted to 0.48 by DiA, compared to the susceptible variety in **Figure 6a**. DiA is aminostyryl dye insoluble in water but fluoresces upon insertion into membranes or when diluted in organic solvent (ThermoFisher, 2025). DiA binds the phospholipid acyl chain of membrane with two alkyl tails to fluoresce in the green region (460/480–580).

In contrast, no green shift is observed between the stained (0.99 in **Figure 6e**) and unstained (1.00 in **Figure 6f**) tolerant variety for LG2 specific to neutral lipids. LG2 is a cell-permeable N-dimethylallylated 3-hydroxyindole-based selective stain for neutral lipids. LG2 does not stain phospholipids and has minimal nonspecific background fluorescence (Kettner and Griehl, 2020).

NIM7 is a naphthalimide-based fluorescent probe that simultaneously tracks lipid droplets (in yellow at 488/530–570 Ex/Em) and lysosome (in red at 561/640–680) in dual color (Zheng et al., 2019). **Figure 6c** indicates some red shift (−0.20, as opposed to −0.07 before staining in **Figure 6d**) by NIM7 staining. However, no distinctive yellow (for lipid droplet)- and red (for lysosome)-stained regions are observable in **Figure 6c**. Lipid droplets are the most hydrophobic sub-structure within cells to store lipids, while lysosomes are acidic (pH 4.5–5) organelles (Zheng et al., 2019). Collectively, **Figure 6** indicates that the bright red fluorescence of the tolerant variety’s vascular bundle by Nile red staining (Uchimiya et al., 2025a) resulted from red emission of phospholipids and other polar lipids (Diaz et al., 2008).

### 3.3 “Net effect” thermal imaging of thawing stalks for tolerant and susceptible varieties

Preceding sections focused on the classification of cold tolerance by metabolites and the visualization of phospholipids in vascular bundles of the tolerant variety containing high fiber. The results suggest variety-specific physiological response to cold stress. Currently, no reliable method exists to monitor the time courses of freeze–thaw cycles for sugarcane exposed to winter freeze in field trials. The number of crack per stalk is visually counted during the freeze trial (Uchimiya et al., 2025a) without monitoring the temperature changes leading to the stalk injury. A thermal camera simultaneously collects RGB and thermal (<0.06 °C resolution) images of sugarcane stalk composed of various components inducing thermal insulation. Amylopectin is the primary (≈80%) component of starch granule in sugarcane (Zhou et al., 2010). Higher amylopectin (over amylose) leads to higher thermal insulation, lower viscosity, and lower mechanical strength (Han et al., 2023). A thermal camera was used to monitor the ripening of avocado in a controlled laboratory experiment (Soesiladi Esti et al., 2024). An aerial thermal camera is widely used to monitor canopy temperature (Zhang et al., 2019). One available field experiment on sugarcane used a portable thermal camera to compare a deep learning model against human evaluation of soil available water content (Melo et al., 2022).

**Figure 7:**
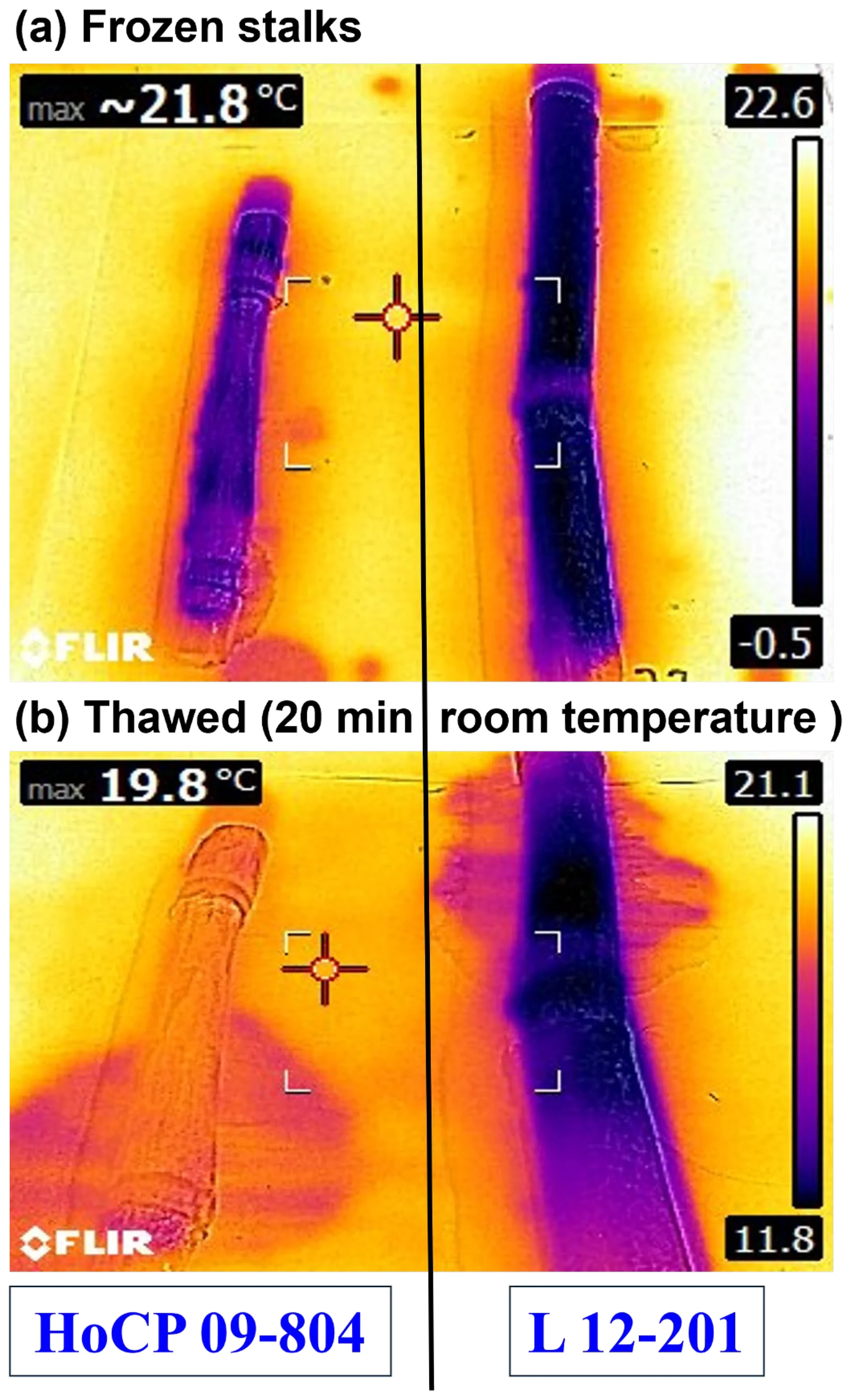
Representative thermal images of frozen **(a)** and thawed **(b)** stalks for HoCP 09-804 (++ reported cold tolerance in **Supplementary Table 1**) and L 12-201 (−) varieties. Darker blue/black indicates lower temperature [towards −0.5 °C limit in **(a)**, indicated by the scale bar], while lighter yellow indicates higher temperature [22.6 °C limit in **(a)**] with <0.06 °C resolution. Tolerant variety (HoCP 09–804 on left) shows faster defrosting indicated by orange/yellow, while susceptible variety (L 12–201 on right) shows internal portions (black areas) with as much as 10 °C lower temperatures.

**Figure 7** presents representative thermal images of frozen (a) and thawed (b, 20 min at room temperature) sugarcane stalks. Temperature is measured at each pixel of the image, and the bar spectrum shows the maximum (22.6 °C in a towards lighter yellow) and minimum (−0.5 °C towards darker blue/black) measured temperatures in each image. When frozen (**Figure 7a**), the susceptible variety (L 12–201 on right) shows black interior portions approaching the minimum measured temperature (−0.5 °C) and higher temperature towards the outer surface of the stalk. Differences between varieties became visible after 20 min of defrosting at room temperature in **Figure 7b**. The cultivar with a higher reported cold tolerance (HoCP09–804 on left) thawed faster than the susceptible one (L 12-201). Representative images in **Figure 7** are influenced by a variety of factors including the diameter and length of imaged talk, similarly to other available ground and aboveground sensors. The freeze–thaw cycle is the cause of stalk rupture and juice deterioration. The variety-dependent thawing rate in **Figure 7** could be used to quantitatively monitor the time courses leading to freeze injury in the field during winter freeze.

## Data Availability

The original contributions presented in the study are included in the article/**Supplementary Material**. Further inquiries can be directed to the corresponding author.

## Supplementary material

Table S1 List of released varieties and experimental lines investigated in this study.
