# Supplementary material for: Multivariate and imaging methods to classify cold tolerance of sugarcane (Saccharum spp. hybrids) cultivars and breeding clones
Source: Front Plant Sci. 2026 Aug 7;17:1882707. doi: 10.3389/fpls.2026.1882707 (PMC13493279; doi:10.3389/fpls.2026.1882707)
Supplement: Supplementary file 1 [file Table1.doc]

Multivariate and Imaging Methods to Classify Cold Tolerance of Sugarcane (*Saccharum* spp. hybrids) Cultivars and Breeding Clones

Minori Uchimiya^*,a^, Collins Kimbeng^b^, Zachary Taylor^b^, Kerstin Höner zu Bentrup^c^, and Kaitlyn Taylor^d^

*^a^United States Department of Agriculture, Agricultural Research Service, Southern Regional Research Center, New Orleans, LA 70124*

*^b^Louisiana State University Agricultural Center, Sugar Research Station, 5755 LSU Ag Rd, St Gabriel, LA 70776*

*^c^Tulane University School of Medicine, Department of Microbiology/Immunology, New Orleans, LA 70112*

*^d^Oak Ridge Institute for Science and Education Research Program at USDA, Oak Ridge, TN 37831*

*Corresponding author fax: (504) 286-4367, phone: (504) 286-4356, email: sophie.uchimiya@usda.gov (M. Uchimiya)

**I.** **List of released varieties and experimental lines investigated in this study.**

**Table S1.** List of commercial varieties (parents, n=33) and clones investigated in this study and described in detail previously (1). Available reports on cold tolerance, male and female parents, and references are provided for commercial varieties (parents). Parents were planted at LSU Dean Lee and sampled in 2023 (1) and 2024 (this study).

| **Parents:** |  |  |  |  |
| --- | --- | --- | --- | --- |
| **genotype** | **tolerance** | **female** | **male** | **Ref** |
| L 99-226 | (-) | CP 89–846 | LCP 81–30 | (2) |
| Ho 05-961 | (++) | CP 83-644 | TUCCP 77-42 | (3) |
| HoCP 04-838 | (+++) | HoCP 85–845 | LCP 85–384 | (4-8) |
| L 15-306 | (++) | CP 83-644 | L 01-283 | (9) |
| Ho 12-615 | (-) | TUCCP77-042 | HoCP 96-540 | (6) |
| HOCP91-552 | (-) | LCP 81–10 | CP 72–356 | (10) |
| TUCCP77-042 | (-) |  |  | (11) |
| L 12-201 | (-) | L 97-128 | HoCP 96-540 | (5, 6) |
| LCP81-010 |  |  |  |  |
| CP83-644  L 98-207 |  | CP 77-310 | CP 77-407 |  |
| L 99-233 | (-) | CP 79-348 | HoCP 91-552 | (12) |
| HoCP 96-540 | (+++) | LCP 86–454 | LCP 85–384 | (11) |
| Ho 07-613 | (-) | HoCP 00-905 | HoCP 96-540 | (13) |
| HoCP 91-555 | (-) | CP 83-644 | LCP 82-094 | (11) |
| L05-457 |  |  |  |  |
| L 01-299 | (+) | L 93-365 | LCP 85–384 | (5, 6, 14) |
| Ho 95-988 |  | CP 86-941 | US89-012 | (15) |
| LCP 85-384 | (+++) | CP 77-310 | CP 77-407 | (16) |
| L11-183 | (++) | HoCP 92–624 | LCP 85–384 | (13) |
| L 14-267 | (+) | HoCP 05-918 | L 01-283 | (5, 6) |
| HOCP92-624 |  |  |  |  |
| HoCP 00-950 | (++) | HoCP 93–750 | HoCP 92–676 | (13) |
| N27 |  |  |  |  |
| L06-001 |  |  |  |  |
| HoCP09-804 | (++) | HoCP 02–625 | HoCP 01–523 | (5, 6, 17) |
| L 03-371 | (-) | CP 83-644 | LCP 82-89 | (13) |
| HoCP 85-845 | (+++) | CP 72-370 | CP 77-403 | (11) |
| HoCP14-802 |  |  |  |  |
| L 97-128 | (+++) | LCP 81–10 | LCP 85–384 | (18) |
| HoCP 14-885 | (+) | HoCP05-920 | Ho 07-613 | (5) |
| HoL 15-508 | (+) | CP 83-644 | L 01-283 | (9) |
| Ho 13-739 | (++) | Ho 06-9610 | HoCP04-838 | (5, 6) |

**2024 final on-station clones at LSU St. Gabriel and their parents. Highlighted varieties are L 01-299 (+ cold tolerance in above table), HoCP09-804 (++), L 14-267 (+), and HoCP 14-885 (+) commercial varieties used as checks in the breeding program. The list below contains 70 total entries for 31 clones with replicate (n=62 for clones) and n=8 for 3 checks**:

| **VARIETY** | **FEMALE** | **MALE** |
| --- | --- | --- |
| 2023156 | LCP81-010 | HO11-9406 |
| 2023153 | HOCP14-867 | HO11-532 |
| 2023165 | HOCP92-624 | HO11-9406 |
| 2023143 | L14-273 | L01-299 |
| 2023138 | HOCP14-867 | HO11-532 |
| 2023142 | L11-187 | HOCP97-609 |
| 2023157 | HOCP92-624 | L99-226 |
| 2023145 | L05-457 | HOCP14-802 |
| 2023144 | HO09-827 | HO11-532 |
| 2023162 | L05-457 | HOCP14-802 |
| 2023166 | L05-457 | L99-233 |
| 2023146 | L13-243 | 18P12 |
| 2023148 | HO09-827 | L06-001 |
| 2023158 | HOCP92-624 | L99-226 |
| 2023139 | HO09-827 | L06-001 |
| 2023152 | L15-337 | L09-099 |
| 2023142 | L11-187 | HOCP97-609 |
| 2023160 | HO13-739 | HOL15-501 |
| 2023136 | L05-457 | L99-233 |
| 2023153 | HOCP14-867 | HO11-532 |
| 2023157 | HOCP92-624 | L99-226 |
| 2023145 | L05-457 | HOCP14-802 |
| 2023141 | HO09-827 | HO11-532 |
| 2023159 | HO09-827 | L06-001 |
| 2023147 | L05-457 | HOCP14-802 |
| 2001299 | L 93–365 | LCP 85-384 |
| 2023140 | HO11-573 | HOCP14-826 |
| 2009804 | HoCP 02–625 | HoCP 01–523 |
| 2023139 | HO09-827 | L06-001 |
| 2014267 | HoCP 05-918 | L 01-283 |
| 2023158 | HOCP92-624 | L99-226 |
| 2014885 | HoCP05-920 | Ho 07-613 |
| 2023154 | HO09-827 | L06-001 |
| 2023136 | L05-457 | L99-233 |
| 2023150 | L05-457 | HOCP14-802 |
| 2023159 | HO09-827 | L06-001 |
| 2023138 | HOCP14-867 | HO11-532 |
| 2023135 | HOCP14-867 | HO11-532 |
| 2023147 | L05-457 | HOCP14-802 |
| 2001299 | L 93–365 | LCP 85-384 |
| 2023143 | L14-273 | L01-299 |
| 2009804 | HoCP 02–625 | HoCP 01–523 |
| 2023151 | L94-433 | 18P15 |
| 2014267 | HoCP 05-918 | L 01-283 |
| 2023144 | HO09-827 | HO11-532 |
| 2014885 | HoCP05-920 | Ho 07-613 |
| 2023162 | L05-457 | HOCP14-802 |
| 2023148 | HO09-827 | L06-001 |
| 2023152 | L15-337 | L09-099 |
| 2023163 | L14-282 | L01-299 |
| 2023137 | L07-057 | 18P1 |
| 2023135 | HOCP14-867 | HO11-532 |
| 2023164 | LCP81-010 | HO11-9406 |
| 2023149 | L05-457 | L99-233 |
| 2023160 | HO13-739 | HOL15-501 |
| 2023155 | L05-457 | 18P2 |
| 2023151 | L94-433 | 18P15 |
| 2023141 | HO09-827 | HO11-532 |
| 2023156 | LCP81-010 | HO11-9406 |
| 2023165 | HOCP92-624 | HO11-9406 |
| 2023140 | HO11-573 | HOCP14-826 |
| 2023163 | L14-282 | L01-299 |
| 2023149 | L05-457 | L99-233 |
| 2023154 | HO09-827 | L06-001 |
| 2023146 | L13-243 | 18P12 |
| 2023150 | L05-457 | HOCP14-802 |
| 2023164 | LCP81-010 | HO11-9406 |
| 2023137 | L07-057 | 18P1 |
| 2023166 | L05-457 | L99-233 |
| 2023155 | L05-457 | 18P2 |

**References**
